# Comparative study of classification algorithms for immunosignaturing data

**DOI:** 10.1186/1471-2105-13-139

**Published:** 2012-06-21

**Authors:** Muskan Kukreja, Stephen Albert Johnston, Phillip Stafford

**Affiliations:** 1 Center for Innovations in Medicine, Biodesign Institute, Arizona State University, Tempe, AZ 85281, USA

**Keywords:** Immunosignature, Random peptide microarray, Data mining, Classification algorithms, Naïve Bayes

## Abstract

**Background:**

High-throughput technologies such as DNA, RNA, protein, antibody and peptide microarrays are often used to examine differences across drug treatments, diseases, transgenic animals, and others. Typically one trains a classification system by gathering large amounts of probe-level data, selecting informative features, and classifies test samples using a small number of features. As new microarrays are invented, classification systems that worked well for other array types may not be ideal. Expression microarrays, arguably one of the most prevalent array types, have been used for years to help develop classification algorithms. Many biological assumptions are built into classifiers that were designed for these types of data. One of the more problematic is the assumption of independence, both at the probe level and again at the biological level. Probes for RNA transcripts are designed to bind single transcripts. At the biological level, many genes have dependencies across transcriptional pathways where co-regulation of transcriptional units may make many genes appear as being completely dependent. Thus, algorithms that perform well for gene expression data may not be suitable when other technologies with different binding characteristics exist. The immunosignaturing microarray is based on complex mixtures of antibodies binding to arrays of random sequence peptides. It relies on many-to-many binding of antibodies to the random sequence peptides. Each peptide can bind multiple antibodies and each antibody can bind multiple peptides. This technology has been shown to be highly reproducible and appears promising for diagnosing a variety of disease states. However, it is not clear what is the optimal classification algorithm for analyzing this new type of data.

**Results:**

We characterized several classification algorithms to analyze immunosignaturing data. We selected several datasets that range from easy to difficult to classify, from simple monoclonal binding to complex binding patterns in asthma patients. We then classified the biological samples using 17 different classification algorithms. Using a wide variety of assessment criteria, we found ‘Naïve Bayes’ far more useful than other widely used methods due to its simplicity, robustness, speed and accuracy.

**Conclusions:**

‘Naïve Bayes’ algorithm appears to accommodate the complex patterns hidden within multilayered immunosignaturing microarray data due to its fundamental mathematical properties.

## Background

Serological diagnostics have received increasing scrutiny recently [1,2] due to their potential to measure antibodies rather than low-abundance biomarker molecules. Antibodies avoid the biomarker dilution problem and are recruited rapidly following infection, chronic, or autoimmune episodes, or exposure to cancer cells. Serological diagnostics using antibodies have the potential to reduce medical costs and may be one of the few methods that allow for true presymptomatic detection of disease. For this reason, our group has pursued immunosignaturing for its ability to detect the diseases early and with a low false positive rate. The platform consists of a peptide microarray with either 10,000 or 330,000 peptides per assay. This microarray is useful with standard mathematical analysis, but for a variety of reasons, certain methods of classification enable the best accuracy [3,4]. Classification methods differ in their ability to handle high or low numbers of features, the feature selection method, and the features’ combined contribution to a linear, polynomial, or complex discrimination threshold. Expression microarrays are quite ubiquitous and relevant to many biological studies, and have been used often when studying classification methods. However, immunosignaturing microarrays may require that we change our underlying assumptions as we determine the suitability of a particular classifier.

In order to establish the question of classification suitability, we examine a basic classification algorithm, Linear Discriminant Analysis (LDA). LDA is widely used in analyzing biomedical data in order to classify two or more disease classes [5-8]. One of the most commonly used high-throughput analytical methods is the gene expression microarray. Probes on an expression microarray are designed to bind to a single transcript, splice variant or methy variant of that transcript. These one-on-one interactions provide relative transcript numbers and cumulatively help to define high-level biological pathways. LDA uses these data to define biologically relevant classes based on the contribution of differentially expressed genes. This method often uses statistically identified features (gene transcripts) that are different from one condition to another. LDA can leverage coordinated gene expression to make predictions based on a fundamental biological process. The advantage of this method is that relatively few features are required to make sweeping predictions. When features change sporadically or asynchronously, the discriminator predictions are adversely affected. This causes low sensitivity in exchange for occasionally higher discrimination. Tree-based methods use far more features to obtain a less biased but less sensitive view of the data. These methods can partition effects even if the effect sizes vary considerably. This approach can be more useful than frequentist approaches where it is important to maintain partitions in discreet groups.

Immunosignaturing has its foundations in both phage display and peptide microarrays. Most phage display methods that use random-sequence libraries also use fairly short peptides, on the order of 8–11 amino acids [9]. Epitope microarrays use peptides in the same size range, but typically far fewer total peptides, on the order of hundreds to thousands [10]. Each of these methods assumes that a single antibody binds to a single peptide, which is either detected by selection (phage display) or by fluorescent secondary antibody (epitope microarray). Immunosignaturing uses long 20-mer random-sequence peptides that have potentially 7 or more possible linear epitopes per peptide. Although immunosignaturing must make do with only 10,000 to ~300,000 peptides, the information content derived from partial binding makes these data useful in ways quite different from phage display [11-15].

**Figure 1:**
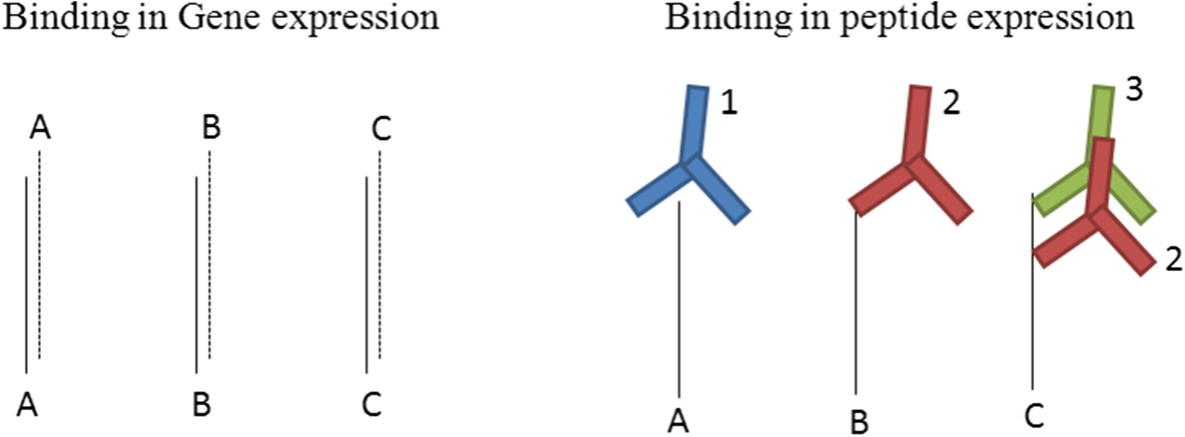
**One-to-one correspondence found in gene expression microarrays is not observed for the immunosignaturing arrays.** We propose that a single peptide may bind numerous antibodies, and have shown that a single antibody can bind hundreds of different peptides.

The complexity in analysis arises from the many-to-many relationship between peptide and antibody (Figure 1). This relationship imposes a particular challenge for classification because a simple one-to-one relationship between probe and target, idiomatic for gene expression microarrays, allows a coherent contribution of many genes that behave coordinately based on biological stimuli. That idiom is broken for immunosignaturing microarrays, where each peptide may bind a number of different antibodies and every antibody might bind a number of peptides. Unless disease-specific antibodies find similar groups of peptides across individuals, very little useful information is available to the classifier. The aim of this work is to assess the performance of various classification algorithms on immunosignaturing data.

We have considered 17 diverse data mining classification methods. For feature selection, we used a simple *t*-test when we examined two classes, and a fixed-effects 1-way ANOVA for multiple classes with no post-hoc stratification. We have assessed these algorithms’ ability to handle increasing numbers of features by providing four different sets of peptides with increasing p-value cutoff. The four levels include from 10 (minimum) to >1000 (maximum) peptides. Each algorithm is thus tested under conditions that highlight either synergistic or antagonistic effects as the feature numbers increase.

## Methods

### Technology

A peptide microarray described previously [11-15] was used to provide data for analysis. Two different sets of 10,000 random peptide sequences are tested. The two peptide sets are non-overlapping and are known as CIM10Kv1 and CIM10Kv2. Peptides are printed as in [1].

### Sample processing

Samples consist of sera, plasma or saliva – each produces a suitable distribution of signals upon detection with an anti-human secondary IgG-specific antibody. Samples are added to the microarray at 1:500 dilutions in sample buffer (1xPBS, 0.5% Tween20, 0.5% Bovine Serum Albumin (Sigma, St. Louis, MO)), IgG antibodies are detected through a biotinylated secondary anti human IgG antibody (Novus anti-human IgG (H + L), Littleton, CO), which binds the primary. Fluorescently labeled streptavidin is used to label the secondary antibodies and scanned with an Agilent ‘C’ laser scanner in single-color mode. 16-bit images are processed using GenePix Pro 8, which provides the tabular information for each peptide in a continuous value ranging from 0–65,000. Four unique data sets have been used in this analysis, 2 run on the CIM10Kv1 and 2 on the CIM10Kv2. Each individual sample was run in duplicate; replicates with >0.8 Pearson correlation coefficient were considered for analysis.

### Datasets

Center for Innovations in Medicine, Biodesign Institute, Arizona State University has an existing IRB 0912004625, which allows analysis of blinded samples from collaborators.

a.) Type 1 diabetes data set: This dataset contains 80 sera samples (41 controls and 39 type 1 diabetes children ages 6 to 13). These samples were tested on the CIM10kV1microarrays.

b.) Alzheimer’s disease data set: This dataset contains 23 samples (12 controls and 11 Alzheimer’s disease subjects). These were tested on the CIM10kV2 microarrays.

c.) Antibodies dataset: This dataset contains 50 samples and has 5 groups monoclonal antibodies, arbitrarily arranged. All monoclonals were raised in mouse, and use the same secondary detection antibody. Samples were run on the CIM10kV1 microarrays.

d.) Asthma dataset: This dataset consists of 47 unique samples containing serum from patients with 4 distinct classes, corresponding to the household environment. Condition A consists of 12 control subjects who had no environmental stimuli. Condition B consists of 12 subjects who had stimuli but no asthma-related symptoms. Condition C consists of 11 subjects who had no stimuli but with clinical asthma. Condition D consists of 12 subjects who have both stimuli and clinical asthma. Samples were tested on the CIM 10 kV2 microarrays. Asthma datasets were been analyzed by considering all four conditions using ANOVA in order to study the combined effect of stimuli and asthma on subjects and then by considering pair wise comparison of condition A vs. B, A vs. C, and B vs. D.

### Data preprocessing, normalization and feature selection

The 16-bit tiff images from the scanned microarrays were imported into GenePix Pro 6.0 (Molecular Devices, Santa Clara, CA). Raw tabular data were imported into Agilent’s GeneSpring 7.3.1 (Agilent, Santa Clara, CA). Data were median normalized per array and log_10_ transformed. For feature selection we used Welch-corrected *T*-test with multiple tested (FWER = 5%). For multiple groups (Antibody and Asthma datasets) we used 1-way fixed-effects ANOVA.

### Data mining classification algorithms

Four distinct peptide features are chosen for the comparison study. For each analysis, peptides are selected by *t*-test or ANOVA across biological classes, with 4 different p-value cutoffs. Cutoffs were selected to obtain roughly equivalent sized feature sets to assess the ability of each algorithm to process sparse to rich feature sets. Once the significant features were collected, data was imported into WEKA [16] for classification. The algorithms themselves spanned a wide variety of classifiers including Bayesian, regression based methods, meta-analysis, clustering, and tree based approaches.

We obtained accuracy from each analysis type using leave-one-out cross-validation. We obtained a list of *t*-test or ANOVA-selected peptides at each stringency level. The highest stringency uses peptides with p-values in the range of 10^-5^ to 10^-10^ and contains the least ‘noise’. The less-stringent second set uses p-values approximately 10-fold higher than the most stringent. The third contains the top 200 peptides and the forth contains ~1000 peptides at p < 0.05. Although different numbers of peptides are used for each dataset, each peptide set yields the same general ability to distinguish the cognate classes. The WEKA default setting of parameters were used for every algorithm to avoid bias and over fitting. These default parameters are taken from the cited papers listed below for each algorithm. Brief details of default parameters and algorithms are listed

I. Naïve Bayes: Probabilistic classifier based on Bayes theorem. Numeric estimator precision values are chosen based on analysis of the training data. In the present study, normal distribution was used for numeric attributes rather than kernel estimator [17].

II. Bayes net: Probabilistic graphical model that represents random variables and conditional dependencies in the form of a directed acyclic graph. A Simple Estimator algorithm has been used for finding conditional probability tables for Bayes net. A K2 search algorithm was used to search network structure [18,19].

III. Logistic Regression (Logistic R.): A generalized linear model that uses logistic curve modeling to fit the probabilistic occurrence of an event[20]. The Quasi-Newton method is used to search for optimization. 1x10^8^ has been used for ridge values in the log likelihood calculation [21].

IV. Simple Logistic: Classifier for building linear logistic regression models. For fitting the logistic model ‘LogitBoost’, simple regression functions are used. Automatic attribute selection is obtained by cross validation of the optimal number of ‘LogitBoost’ iterations [22]. Heuristic stop parameter is set at 50. The number of maximum iterations for LogitBoost has been set to 500.

V. Support Vector Machines (SVM): A non-probabilistic binary linear classifier that constructs one or more hyper planes to be can be used for classification. For training support vector classes, John Platt’s sequential minimal optimization algorithm was used which replaces all missing values [23]. Here multiclass problems are used using pair-wise classification. The complexity parameter is set to 1. Epsilon for round off error is set to 1x10*^-12^. PolyKernel is the set to be kernel. The tolerance parameter is set to 0.001 [24,25].

VI. Multilayer Perceptron (MLP): A supervised learning technique with a feed forward artificial neural network through back-propagation that can classify non-linearly separable data [26,27]. The learning rate is set to 0.3 and momentum applied during updating weights is set to 0.2. The validation threshold use to terminate the validation testing is set to 20.

VII. K nearest neighbors (KNN): Instance based learning or lazy learning which trains the classifier function locally by majority note of its neighboring data points. Linear NN Search algorithm is used for search algorithm [28,29]. K is set to 3.

VIII. K Star: Instance based classifier that uses similarity function from the training set to classify test set. Missing values are averaged by column entropy curves and global blending parameter is set to 20 [30].

IX. Attribute Selected Classifier (ASC): ‘Cfs subset’ evaluator is used during the attribute selection phase to reduce the dimension of training and test data. The ‘BestFit’ search method is invoked after which J48 tree classifier is used [31].

X. Classification via clustering (K means): Simple k means clustering method is used where k is set to the number of classes in the data set [32]. Euclidean distance was used for evaluation with 500 iterations.

XI. Classification via Regression (M5P): Regression is a method used to evaluate the relationship between dependent and independent variables through an empirically determined function. The M5P base classifier is used which combines conventional decision tree with the possibility of linear regression at the nodes. The minimum number of instances per leaf node is set to 4 [33].

XII. Linear Discriminant Analysis (LDA): Prevalent classification technique that identifies the combination of features that best characterizes classes through linear relationships. Prior probabilities are set to uniform and the model as homoscedastic.

XIII. Hyper Pipes: Simple, fast classifier that counts internally defined attributes for all samples and compares the number of instances of each attribute per sample. Classification is based on simple counts. Works well when there are many attributes [34].

XIV. VFI: Voting feature interval classifier is a simple heuristic attribute-weighting scheme. Intervals are constructed for numeric attributes. For each feature per interval, class counts are recorded and classification is done by voting. Higher weight is assigned to more confident intervals. The strength of the bias towards more confident features is set to 0 [35].

XV. J48: Java implementation of C4.5 algorithm. Based on the Hunt’s algorithm, pruning takes place by replacing internal node with a leaf node. Top-down decision tree/voting algorithm [36]. 0.25 is used for the confidence factor. No Laplace method for tree smoothing [37].

XVI. Random Trees: A tree is grown from data that has K randomly chosen attributes at each node. It does not perform pruning. K-value (log_2_ (number of attributes) + 1) is set at zero. There is no depth restriction. The minimum total weight per leaf is set to 1 [34].

XVII. Random Forest (R. Forest): Like Random Tree, the algorithm constructs a forest of random trees [38] with locations of attributes chosen at random. It uses an ensemble of unprune decision trees by a bootstrap sample using training data. There is no restriction on the depth of the tree; number of tress used is 100.

### Time performance

CPU time was calculated for every algorithm at the four different significance levels. This time was measured on a standard PC (Intel dual core, 2.2 GHz 3 Gb RAM) that was completely dedicated to WEKA. To measure CPU time, open source jar files from WEKA were imported to Eclipse where the function ‘time ()’ was invoked prior to running the classification including the time required for cross validation. Most Windows 7 services were switched off; the times reported were an average of 5 different measurements.

## Results

### Overall performance accuracy of classification algorithms over all data sets

For each dataset, accuracies are measured at four levels (top 10, 50,200, 1000 peptides) at various levels of significance. Overall average performance measure is calculated for each algorithm for a given data set. Table 1 shows the overall average percentage score for each algorithm calculated by averaging accuracy, specificity, sensitivity and area under ROC curve under all levels of significance. Scores >90% are marked in bold. MLP algorithm did not finish due to huge memory requirements on last level of significance and is averaged based on first three levels of significance. For type 1 diabetes, Alzheimer’s and antibodies dataset, >6 algorithms scored >90% average score. Overall, Naïve Bayes had the highest average score (90.4%) and was always among top 3 algorithms among all datasets.

**Table 1:** Overall performance measure of classification algorithms on datasets

**Algorithms**	**T1D**	**Az**	**Ab**	**Asthma**	**A & B**	**A & C**	**B & D**	**Avg.**	**Rank**
**Naïve Bayes**	**92.0**	**93.4**	**91.5**	77.7	**90.8**	**93.5**	**93.6**	**90.4**	**1**
**MLP**	**90.1**	**92.7**	**90.2**	71.1	84.7	**92.7**	89.3	87.3	**2**
**SVM**	**91.6**	88.0	**90.7**	71.3	86.1	88.4	**93.1**	87.0	**3**
**VFI**	**90.5**	**92.2**	75.5	62.6	87.7	**93.4**	**92.7**	84.9	**4**
**Hyper Pipes**	89.8	89.7	81.3	62.3	82.0	86.6	87.8	82.8	**5**
**R. Forest**	**91.5**	82.4	**93.3**	62.8	80.6	81.4	81.1	81.9	**6**
**Bayes Net**	**90.3**	87.7	**92.5**	53.9	80.2	83.2	85.1	81.8	**7**
**K-means**	88.3	**91.8**	80.7	59.6	77.8	83.3	83.6	80.7	**8**
**Logistic R.**	**90.6**	**93.3**	60.4	50.7	81.5	84.8	**90.7**	78.9	**9**
**SLR**	**92.2**	71.8	**90.1**	72.2	65.0	68.5	84.7	77.8	**10**
**KNN**	**91.4**	81.5	52.5	55.8	87.5	75.7	89.0	76.2	**11**
**K star**	81.9	**90.7**	89.4	53.5	64.3	68.8	70.7	74.2	**12**
**M5P**	85.1	58.7	83.2	60.0	75.2	73.4	79.6	73.6	**13**
**J48**	80.3	69.7	78.4	48.7	70.6	68.4	76.7	70.4	**14**
**Random Tree**	83.8	71.7	76.2	52.9	69.3	60.8	75.0	70.0	**15**
**ASC**	76.8	70.0	77.9	43.1	72.0	63.1	76.7	68.5	**16**
**LDA**	69.7	52.0	89.1	70.8	62.8	69.7	52.6	66.7	**17**

T1D: Type 1 diabetes datasets, Az: Alzehemer’s dataset, Ab: Antibodies dataset. Table showing algorithms overall performance in each datasets based on average score. Score >90% are marked in bold. Naïve Bayes scored the overall highest average score of 90.4%.

### Performance accuracy of classification algorithms at different levels of significance over all data sets

For each data set, different levels of significance are chosen to measure the performance accuracy of each algorithm. These levels contain approximately equal number of peptides for each data set. The first level contains 10 peptides selected from the *t*-test (lowest p value) and hence contains the least noise. Next, approximately 50 peptides, 200 peptides and 1000 peptides were chosen for the other three levels.

Tables 2, 3, 4, 5, 6, 7, 8 shows 4 different performance measures (accuracy, specificity, sensitivity and area under ROC curve) at different levels of significance over 7 datasets. For the Asthma dataset, we considered all conditions A-D together, then performed the pair-wise comparisons of condition A and B, condition A and C, and condition B and D at three different levels of significance. Measures >90% are marked in bold. For the diabetes dataset, 9 algorithms achieved >90% score. For Alzheimer’s and the Antibodies dataset, 6 algorithms achieved >90% score. Naïve Bayes scored 100% in all 4 measures at the first level of significance in the Alzheimer’s dataset and scored 91.5% average score on the Antibodies dataset. For the Asthma datasets, the highest score was <80%. Only Naïve Bayes had >90% specificity for more than one level of significance. For two conditions in Asthma datasets, Naïve Bayes and VFI scored >90% average score.

**Table 2:** Performance measures of data mining algorithm at different levels of significance over Type 1 diabetes dataset

**SIGNIFICANCE**	**p < 5 x 10**^**-13**^				**p < 5 x 10**^**-10**^				**p < 5 x 10**^**-7**^				**p < 5 x 10**^**-4**^				
**Algorithm**	**Acc.**	**Sp**	**Sn**	**AUC**	**Acc.**	**Sp**	**Sn**	**AUC**	**Acc.**	**Sp**	**Sn**	**AUC**	**Acc**	**Sp**	**Sn**	**AUC**	**Avg.**
**SLR**	87.5	85.0	89.7	**0.93**	**92.5**	**90.2**	**94.9**	**0.97**	**92.5**	**92.0**	**92.0**	**0.96**	**92.5**	**90.0**	**94.9**	**0.96**	**92.2**
**Naïve Bayes**	**90.0**	85.4	**95.0**	**0.97**	**91.3**	**90.2**	**92.3**	**0.98**	**92.5**	**90.2**	**95.0**	**0.96**	89.0	85.4	**92.3**	**0.92**	**92.0**
**SVM**	88.8	82.9	**94.9**	0.89	**90.0**	82.9	**97.4**	**0.90**	**93.8**	**90.2**	**97.4**	**0.93**	**93.8**	**92.7**	**94.9**	**0.94**	**91.6**
**R. Forest**	87.5	87.8	87.2	**0.96**	**92.5**	**90.2**	**94.9**	**0.97**	**91.5**	87.8	**94.9**	**0.97**	88.8	85.4	**92.3**	**0.94**	**91.5**
**KNN**	**92.5**	**90.2**	**94.9**	**0.95**	**95.0**	**92.7**	**97.4**	**0.96**	**90.0**	85.4	**94.9**	**0.93**	85.0	80.5	89.7	**0.90**	**91.4**
**Logistic. R**	86.3	87.8	84.6	0.82	**92.5**	**90.2**	**94.9**	**0.97**	**92.5**	**92.7**	**97.4**	**0.97**	87.5	**92.7**	82.1	**0.92**	**90.6**
**VFI**	87.5	82.9	**92.3**	**0.95**	**92.5**	**90.2**	**94.9**	**0.97**	88.8	85.4	**92.3**	**0.95**	87.5	82.9	**92.3**	**0.92**	**90.5**
**Bayes Net**	**91.3**	**90.2**	**92.3**	**0.97**	**90.0**	85.4	**94.9**	**0.98**	**90.0**	85.4	**94.9**	**0.95**	83.8	78.0	89.7	0.89	**90.3**
**MLP**	80.0	80.5	79.5	0.89	**91.3**	**90.2**	**92.3**	**0.98**	**93.8**	**90.2**	**97.4**	**0.99**	dnf	dnf	dnf	dnf	90.1*
**Hyper Pipes**	87.5	**90.2**	84.6	**0.96**	**91.3**	**90.2**	**92.3**	**0.97**	**90.0**	**90.2**	89.7	**0.95**	83.8	**92.7**	74.4	**0.92**	89.8
**K-means**	**91.3**	82.9	**100**	**0.92**	**90.0**	82.9	**97.4**	**0.90**	86.3	78.0	**94.9**	0.87	85.0	75.6	**94.9**	0.85	88.3
**M5P**	88.8	85.4	**92.3**	**0.94**	85.0	80.5	89.7	**0.94**	81.3	78.0	84.6	0.87	78.8	73.2	84.6	0.85	85.1
**Random Tree**	85.0	87.8	82.1	0.85	78.8	75.6	82.1	0.79	87.5	85.4	89.7	0.88	83.8	85.4	82.1	0.84	83.8
**K star**	87.5	87.8	87.2	**0.96**	**91.3**	85.4	**97.4**	0.98	**90.0**	85.4	**94.9**	**0.97**	53.8	**100**	5.1	0.54	81.9
**J48**	86.3	85.4	87.2	0.79	81.3	82.9	79.5	0.83	78.8	82.9	74.4	0.72	80.0	85.4	74.4	0.73	80.3
**ASC**	86.3	85.4	87.2	0.79	80.0	82.9	76.9	0.80	80.0	87.8	71.8	0.78	66.3	80.5	51.3	0.55	76.8
**LDA**	88.8	82.9	**94.9**	**0.96**	**91.3**	85.4	**97.4**	0.95	40.0	**96.7**	15.8	0.68	21.3	**94.4**	0.0	0.48	69.7

Acc: Accuracy, Sp: Specificity, Sn: Sensitivity, AUC: Area under ROC curve, Avg: Average score in % for each algorithms, dnf: “Did Not Finish”, * denotes Avg. from 3 significance levels. Measures >90% are marked in bold.

**Table 3:** Performance measures of data mining algorithm at different levels of significance over Alzheimer’s dataset

**SIGNIFICANCE**	**p < 5 x 10**^**-5**^				**p < 5 x 10**^**-4**^				**p < 5 x 10**^**-3**^				**p < 5 x 10**^**-2**^				
**Algorithm**	**Acc.**	**Sp**	**Sn**	**AUC**	**Acc.**	**Sp**	**Sn**	**AUC**	**Acc.**	**Sp**	**Sn**	**AUC**	**Acc**	**Sp**	**Sn**	**AUC**	**Avg.**
**Naïve Bayes**	**100**	**100**	**100**	**1.00**	**91.3**	82.0	**100**	**0.96**	**91.3**	82.0	**100**	**0.96**	86.5	**91.0**	84.0	**0.94**	**93.4**
**Logistic. R**	**95.0**	**90.0**	**100**	**0.99**	**95.7**	**90.0**	**100**	**0.97**	**91.3**	**90.0**	**91.7**	**0.90**	**91.3**	**90.0**	**91.7**	**0.90**	**93.3**
**MLP**	**91.3**	**90.9**	**91.7**	**0.97**	**95.6**	**90.9**	**100**	**0.97**	87.0	**90.9**	83.3	**0.97**	*dnf*	*dnf*	*dnf*	*dnf*	92.7*
**VFI**	**91.3**	**90.9**	**91.7**	0.87	**95.7**	**90.9**	**100**	**0.92**	**91.3**	81.8	**100**	0.89	**91.3**	81.8	**100**	**1.00**	**92.2**
**KNN**	**91.3**	**90.9**	**91.7**	**0.93**	**95.6**	**90.9**	**100**	**0.93**	86.9	**90.9**	83.3	**0.95**	**91.3**	**90.9**	**91.7**	**0.92**	**91.8**
**K-means**	82.6	**100**	66.7	0.83	**91.3**	**90.9**	**100**	**0.91**	**95.7**	**90.9**	**100**	**0.96**	**91.3**	81.8	**100**	**0.90**	**90.7**
**Hyper Pipes**	**91.3**	81.8	**100**	**0.98**	**95.7**	**90.9**	**100**	**0.97**	**91.3**	81.8	**100**	**0.95**	73.9	81.8	66.7	**0.90**	89.7
**SVM**	87.0	**90.9**	83.3	0.87	**95.7**	**90.9**	**100**	**0.95**	82.6	81.8	83.3	0.83	87.0	81.8	**91.7**	0.87	88.0
**Bayes Net**	**91.3**	81.8	**100**	**0.96**	**91.3**	**90.9**	**91.7**	**0.95**	87.0	81.8	**91.7**	0.86	78.3	81.8	75.0	0.84	87.7
**R. Forest**	86.9	81.8	**91.7**	**0.94**	82.6	81.8	83.3	**0.93**	73.9	72.7	75.0	0.89	72.6	81.8	75.0	0.84	82.4
**K star**	**95.7**	**90.9**	**100**	**0.98**	**91.3**	**90.9**	**91.7**	**0.94**	78.2	81.8	75.0	0.86	56.5	18.2	**91.7**	0.64	81.5
**SLR**	86.9	81.8	**91.7**	**0.96**	73.9	72.7	75.0	0.82	60.9	63.6	58.3	0.80	52.2	54.5	50.0	0.69	71.8
		**Random Tree**	78.3	72.7	83.3	0.78	60.9	54.5	66.7	0.61	73.9	63.6	83.3	0.74	73.9	81.8	66.7	0.74	71.7
**ASC**	73.9	63.6	83.3	0.61	68.9	63.6	58.3	0.56	73.9	81.8	66.7	0.75	78.2	63.9	**91.7**	0.61	70.0		
**J48**	73.9	63.6	83.3	0.61	60.9	63.6	58.3	0.56	73.9	81.8	70.0	0.75	78.3	63.6	**91.7**	0.61	69.7		
**M5P**	69.5	54.5	83.3	0.80	52.2	45.5	58.3	0.73	56.5	45.5	66.7	0.43	56.5	36.4	75.0	0.44	58.7		
**LDA**	69.6	72.7	66.7	0.81	34.8	40.0	75.0	0.45	34.8	0.0	**100**	0.30	30.4	**100**	0.0	0.52	52.0		

Acc: Accuracy, Sp: Specificity, Sn: Sensitivity, AUC: Area under ROC curve, Avg: Average score in % for each algorithms, *dnf:* Did not Finish”, * denotes Avg. from 3 significance levels. Measures >90% are marked in bold.

**Table 4:** Performance measures of data mining algorithm at different levels of significance over Antibodies dataset

**SIGNIFICANCE**	**p < 5 x 10**^**-8**^				**p < 5 x 10**^**-7**^				**p < 5 x 10**^**-6**^				**p < 5 x 10**^**-5**^				
**Algorithm**	**Acc.**	**Sp**	**Sn**	**AUC**	**Acc.**	**Sp**	**Sn**	**AUC**	**Acc.**	**Sp**	**Sn**	**AUC**	**Acc**	**Sp**	**Sn**	**AUC**	**Avg.**
**R. Forest**	**90.0**	**93.0**	**90.0**	**0.96**	**90.0**	**91.0**	**90.0**	**0.97**	**92.0**	**94.0**	**92.0**	**0.96**	**94.0**	**96.0**	**94.0**	**0.97**	**93.3**
**Bayes Net**	88.0	**92.0**	88.0	**0.96**	88.0	**91.0**	88.0	**0.96**	**94.0**	**95.0**	**94.0**	**0.95**	**92.0**	**95.0**	**92.0**	**0.96**	**92.5**
**Naïve Bayes**	88.0	**94.0**	88.0	**0.96**	88.0	**94.0**	88.0	**0.96**	88.0	**94.0**	88.0	**0.96**	88.0	**94.0**	88.0	**0.96**	**91.5**
**SVM**	80.0	86.6	80.0	0.86	86.0	89.9	86.0	0.89	**94.0**	**96.6**	**97.0**	**0.95**	**96.0**	**96.9**	**96.0**	**0.96**	**90.7**
**MLP**	80.0	89.8	80.0	**0.91**	86.0	89.9	86.0	**0.96**	**94.0**	**96.6**	**94.0**	**0.99**	*dnf*	*dnf*	*dnf*	*dnf*	90.2*
**SLR**	84.0	**91.6**	84.0	0.89	86.0	83.2	86.0	**0.92**	**90.0**	**93.5**	**90.0**	**0.97**	**92.0**	**95.0**	**92.0**	**0.96**	**90.1**
**KNN**	82.0	**90.7**	82.0	**0.92**	84.0	88.7	84.0	**0.94**	86.0	**91.2**	86.0	**0.95**	**92.0**	**96.4**	**92.0**	**0.95**	89.4
**Logistic R.**	72.0	85.3	72.0	**0.92**	84.0	**90.1**	84.0	**0.93**	**92.0**	**96.4**	**92.0**	**0.98**	**90.0**	**96.1**	**90.0**	**0.98**	89.1
**M5P**	80.0	**91.5**	80.0	**0.92**	76.0	87.4	76.0	**0.90**	78.0	89.4	78.0	**0.91**	74.0	85.4	74.0	0.89	83.2
**Hyper Pipes**	64.0	83.6	64.0	**0.90**	72.0	84.9	72.0	**0.90**	80.0	87.5	**80.0**	**0.92**	80.0	87.1	80.0	**0.93**	81.3
**K star**	88.0	**93.4**	88.0	**0.94**	**94.0**	**97.2**	**94.0**	**0.95**	82.0	**91.8**	**82.0**	**0.93**	20.0	**90.2**	20.8	0.68	80.7
**J48**	80.0	**92.5**	80.0	0.86	72.0	87.0	72.0	0.87	70.0	87.6	70.0	0.79	64.0	86.1	64.0	0.77	78.4
**ASC**	82.0	**91.7**	82.0	0.87	72.0	82.9	72.0	0.82	70.0	87.8	70.0	0.76	64.0	88.5	64.0	0.75	77.9
**Random Tree**	72.0	**90.3**	72.0	0.81	64.0	82.1	64.0	0.73	68.0	87.7	68.0	0.78	74.0	89.7	74.0	0.82	76.2
**VFI**	72.0	88.5	72.0	0.86	64.0	**91.9**	64.0	0.85	58.0	**94.7**	58.0	0.86	52.0	**94.5**	52.0	0.89	75.5
**LDA**	68.0	84.5	68.0	0.88	40.0	81.1	40.0	0.71	42.0	89.7	48.8	0.54	20.0	88.4	25.0	0.58	60.4
**K means**	46.0	68.7	46.0	0.57	46.0	68.7	46.0	0.57	40.0	68.1	40.0	0.54	40.0	68.1	40.0	0.54	52.5

Acc: Accuracy, Sp: Specificity, Sn: Sensitivity, AUC: Area under ROC curve, Avg: Average score in % for each algorithms, *dnf:* Did not Finish”, * denotes Avg. from 3 significance levels. Measures >90% are marked in bold.

**Table 5:** Performance measures of data mining algorithm at different levels of significance over Asthma dataset 4 classes

**SIGNIFICANCE**	**p < 5 x 10**^**-5**^				**p < 5 x 10**^**-4**^				**p < 5 x 10**^**-3**^				**p < 5 x 10**^**-2**^				
**Algorithm**	**Acc.**	**Sp**	**Sn**	**AUC**	**Acc.**	**Sp**	**Sn**	**AUC**	**Acc.**	**Sp**	**Sn**	**AUC**	**Acc**	**Sp**	**Sn**	**AUC**	**Avg.**
**Naïve Bayes**	61.7	87.2	61.7	0.82	68.1	89.3	68.1	0.86	72.3	**90.8**	72.3	0.87	70.2	**90.0**	70.2	0.86	77.7
**SLR**	57.5	85.8	57.4	0.80	57.4	85.6	57.4	0.81	72.3	**90.7**	72.3	0.85	55.3	86.1	55.3	0.76	72.2
**SVM**	55.3	86.2	55.3	0.77	55.3	86.2	55.3	0.77	61.7	87.2	61.7	0.82	66.0	87.6	66.0	0.81	71.3
**MLP**	55.3	86.1	55.3	0.82	53.2	84.6	53.2	0.80	63.8	87.8	63.8	0.88	*dnf*	*dnf*	*dnf*	*dnf*	71.1*
**Logistic R.**	48.9	87.0	48.9	0.78	53.2	84.4	53.2	0.79	59.6	86.4	59.6	0.84	68.0	89.2	68.1	0.86	70.8
**R. Forest**	48.9	86.9	48.9	0.77	48.9	86.9	48.9	0.77	46.8	81.1	46.8	0.75	40.4	80.0	40.4	0.71	62.8
**VFI**	48.9	82.8	48.9	0.66	48.9	82.9	48.9	0.67	51.0	83.6	51.1	0.69	46.8	81.9	46.8	0.77	62.6
**Hyper Pipes**	51.1	83.4	51.1	0.72	53.2	84.0	53.2	0.70	46.8	71.8	46.8	0.74	42.6	80.3	42.0	0.75	62.3
**M5P**	48.9	82.8	48.9	0.79	55.3	86.1	55.3	0.81	42.5	81.0	42.6	0.68	27.6	75.8	27.7	0.57	60.0
**KNN**	42.5	87.1	42.6	0.69	46.8	86.6	46.8	0.67	44.6	88.0	44.7	0.69	36.2	79.7	36.2	0.67	59.6
**K means**	40.4	81.9	40.4	0.60	46.8	82.2	46.8	0.65	42.6	80.7	42.6	0.62	34.0	78.0	34.0	0.56	55.8
**Bayes Net**	38.3	79.3	38.3	0.56	36.2	77.8	36.2	0.56	44.7	81.4	44.7	0.63	36.2	77.6	36.2	0.60	53.9
**K star**	48.9	83.0	48.9	0.70	38.3	79.4	38.3	0.63	36.2	79.4	36.2	0.62	23.4	76.4	23.4	0.49	53.5
**Random Tree**	29.8	76.6	29.8	0.53	40.4	80.2	40.4	0.60	38.3	79.5	38.3	0.59	40.4	80.2	40.4	0.60	52.9
**LDA**	53.2	84.4	53.2	0.80	27.7	80.0	32.5	0.57	8.5	86.5	16.7	0.56	14.9	83.6	23.3	0.53	50.7
**J48**	27.7	75.4	27.7	0.52	27.7	75.9	27.7	0.49	42.6	80.8	42.6	0.58	31.9	77.1	31.9	0.52	48.7
**ASC**	27.7	76.0	27.7	0.52	19.2	71.8	19.1	0.46	29.8	76.7	29.8	0.52	21.2	74.8	21.3	0.45	43.1

Acc: Accuracy, Sp: Specificity, Sn: Sensitivity, AUC: Area under ROC curve, Avg: Average score in % for each algorithms, *dnf:* Did not Finish”, * denotes Avg. from 3 significance levels. Measures >90% are marked in bold.

**Table 6:** Performance measures of data mining algorithm at different levels of significance on A & B conditions

**SIGNIFICANCE**	**p < 5 x 10**^**-4**^				**p < 5 x 10**^**-3**^				**p < 5 x 10**^**-2**^				
**Algorithm**	**Acc.**	**Sp**	**Sn**	**AUC**	**Acc.**	**Sp**	**Sn**	**AUC**	**Acc.**	**Sp**	**Sn**	**AUC**	**Avg.**
**Naïve Bayes**	87.5	83.3	**91.7**	0.84	**91.7**	83.3	**100**	**0.97**	**91.7**	83.3	**100**	**0.96**	**90.8**
**VFI**	79.2	75.0	83.3	**0.93**	**91.7**	83.3	**100**	**0.95**	87.5	75.0	**100**	**0.90**	87.7
**K means**	87.5	83.3	**91.7**	0.88	**91.7**	83.3	**100**	**0.92**	83.3	75.0	**91.7**	0.83	87.5
**SVM**	83.3	83.3	83.3	0.83	87.5	**91.7**	83.3	0.87	87.5	83.3	**91.7**	0.88	86.1
**MLP**	79.2	83.3	75.0	0.70	**91.7**	**91.7**	**91.7**	**0.95**	*dnf*	*dnf*	*dnf*	*dnf*	84.7*
**Hyper Pipes**	83.3	75.0	**91.7**	**0.91**	83.3	83.3	83.3	**0.93**	70.8	83.3	58.3	0.88	82.0
**Logistic R.**	66.7	83.3	50.0	0.76	**95.8**	**91.7**	**100**	**0.92**	79.2	83.3	75.0	0.85	81.5
**Random Forest**	79.2	83.3	75.0	**0.91**	79.2	75.0	83.3	0.86	79.2	75.0	83.3	0.78	80.6
**Bayes Net**	83.3	75.0	**91.7**	0.87	83.3	83.3	83.3	0.83	75.0	75.0	75.0	0.67	80.2
**KNN**	75.0	83.3	66.7	0.85	75.0	**91.7**	58.3	**0.90**	75.0	**91.7**	58.3	0.84	77.8
**M5P**	75.0	83.3	66.7	0.74	75.0	75.0	75.0	0.79	75.0	75.0	75.0	0.74	75.2
**ASC**	62.5	66.7	58.3	0.65	79.2	83.3	75.0	0.85	70.8	75.0	66.7	0.76	72.0
**J48**	62.5	66.7	58.3	0.65	79.2	83.3	75.0	0.85	66.7	75.0	58.3	0.72	70.6
**Random Tree**	70.8	75.0	66.7	0.70	70.8	75.0	66.7	0.70	66.7	66.7	66.7	0.67	69.3
**SLR**	70.8	75.0	66.7	0.80	66.7	75.0	58.3	0.77	50.0	50.0	50.0	0.60	65.0
**K star**	66.7	**91.7**	41.7	0.83	58.3	**100**	46.7	0.83	50.0	0.0	**100**	0.50	64.3
**LDA**	79.2	83.3	75.0	0.84	61.2	64.5	54.5	0.52	29.2	14.3	**100**	0.56	62.8

Acc: Accuracy, Sp: Specificity, Sn: Sensitivity, AUC: Area under ROC curve, Avg: Average score in % for each algorithms, *dnf:* Did not Finish”, * denotes Avg. from 3 significance levels. Measures >90% are marked in bold.

**Table 7:** Performance measures of data mining algorithm at different levels of significance on A & C conditions

**SIGNIFICANCE**	**p < 5 x 10**^**-4**^				**p < 5 x 10**^**-3**^				**p < 5 x 10**^**-2**^				
**Algorithm**	**Acc.**	**Sp**	**Sn**	**AUC**	**Acc.**	**Sp**	**Sn**	**AUC**	**Acc.**	**Sp**	**Sn**	**AUC**	**Avg.**
**Naïve Bayes**	**91.3**	**91.7**	**91.0**	**0.94**	**96.0**	**100**	**90.9**	**0.99**	**91.3**	**100**	81.8	**0.95**	**93.5**
**VFI**	**95.6**	**100**	**90.0**	**0.97**	**95.6**	**100**	**90.0**	**0.97**	87.0	83.3	**90.0**	**0.95**	**93.4**
**MLP**	86.9	**91.7**	81.8	**0.97**	**95.6**	**100**	**90.9**	**0.98**	*dnf*	*dnf*	*dnf*	*dnf*	92.7*
**SVM**	**95.6**	**100**	**90.9**	**0.96**	**95.7**	**100**	**90.9**	**0.96**	73.9	75.0	72.7	0.74	88.4
**Hyper Pipes**	**95.7**	**100**	**90.9**	**0.99**	82.6	**91.7**	72.7	**0.90**	78.2	83.3	72.7	0.83	86.6
**Logistic R.**	86.0	**91.7**	81.8	**0.96**	**95.7**	**100**	**90.9**	**0.92**	69.6	83.3	54.5	0.76	84.8
**KNN**	**91.3**	**100**	81.8	**0.92**	**91.3**	**100**	81.8	**0.94**	65.2	66.7	63.6	0.72	83.3
**Bayes Net**	**95.7**	**100**	**90.9**	**0.99**	82.6	83.3	81.8	**0.92**	69.6	66.7	72.7	0.64	83.2
**Random Forest**	87.0	83.3	**90.9**	**0.93**	82.6	83.3	81.8	**0.91**	69.5	66.7	72.7	0.75	81.4
**K means**	69.6	83.3	54.5	0.69	**95.7**	**100**	**90.9**	**0.95**	60.9	63.6	63.6	0.63	75.7
**M5P**	**91.3**	**91.7**	**90.9**	0.86	65.2	58.3	72.7	0.72	65.2	58.3	72.7	0.56	73.4
**LDA**	**91.3**	**100**	81.8	**0.97**	65.2	71.7	58.6	0.77	17.4	25.0	**100**	0.52	69.7
**K star**	73.9	**91.7**	54.5	**0.93**	78.2	**100**	54.5	0.82	47.8	0.0	**100**	0.50	68.8
**SLR**	87.0	83.3	**90.9**	0.89	73.9	75.0	72.7	0.74	43.5	41.7	45.5	0.45	68.5
**J48**	69.6	66.7	72.7	0.76	69.6	58.3	81.8	0.77	60.9	58.3	63.6	0.66	68.4
**ASC**	65.6	66.7	72.7	0.76	69.6	66.7	72.7	0.76	47.8	66.7	27.3	0.49	63.1
**Random Tree**	73.9	**91.7**	54.5	0.73	73.9	66.7	81.8	0.74	34.8	33.3	36.4	0.35	60.8

Acc: Accuracy, Sp: Specificity, Sn: Sensitivity, AUC: Area under ROC curve, Avg: Average score in % for each algorithms, *dnf:* Did not Finish”, * denotes Avg. from 3 significance levels. Measures >90% are marked in bold.

**Table 8:** Performance measures of data mining algorithm at different levels of significance on B & D conditions

**SIGNIFICANCE**	**p < 5 x 10**^**-4**^				**p < 5 x 10**^**-3**^				**p < 5 x 10**^**-2**^				
**Algorithm**	**Acc.**	**Sp**	**Sn**	**AUC**	**Acc.**	**Sp**	**Sn**	**AUC**	**Acc.**	**Sp**	**Sn**	**AUC**	**Avg.**
**Naïve Bayes**	**91.7**	**100**	83.3	**0.95**	**91.7**	**91.7**	**91.7**	**0.92**	**95.8**	**91.7**	**100**	**0.98**	**93.6**
**SVM**	**91.7**	**100**	83.3	**0.92**	**91.7**	**91.7**	**91.7**	**0.92**	**95.8**	**100**	**91.7**	**0.96**	**93.1**
**VFI**	87.5	**100**	75.0	**0.93**	**91.7**	**100**	83.3	**0.94**	**95.8**	**100**	**91.7**	**1.00**	**92.7**
**Logistic R.**	79.1	83.3	75.0	**0.92**	**100**	**100**	**100**	**1.00**	87.5	**91.7**	83.3	**0.97**	**90.7**
**MLP**	87.5	**91.7**	83.3	**0.94**	87.5	83.3	**91.7**	**0.96**	*dnf*	*dnf*	*dnf*	*dnf*	89.3*
**K means**	87.5	**91.7**	83.3	0.88	**91.4**	**91.7**	**91.7**	**0.92**	87.5	83.3	**91.7**	0.88	89.0
**Hyper Pipes**	87.5	83.3	**91.7**	0.89	**91.7**	**91.7**	**91.7**	0.87	83.3	75.0	**91.7**	**0.90**	87.8
**Bayes Net**	83.3	83.3	83.3	0.89	87.5	**91.7**	83.3	0.86	83.3	83.3	83.3	0.84	85.1
**SLR**	83.3	83.3	83.3	0.88	79.2	66.7	**91.7**	**0.90**	87.5	**100**	75.0	0.89	84.7
**KNN**	79.2	75.0	83.3	0.80	83.3	83.3	83.3	0.83	87.5	**91.7**	83.3	**0.90**	83.6
**Random Forest**	83.3	83.3	83.3	0.83	79.2	83.3	75.0	0.84	79.2	83.3	75.0	0.81	81.1
**M5P**	87.5	**91.7**	83.3	0.88	79.2	83.3	75.0	0.73	75.0	83.3	66.7	0.69	79.6
**ASC**	**91.7**	**100**	83.3	0.83	75.0	83.3	66.7	0.61	70.8	75.0	66.7	0.64	76.7
**J48**	**91.7**	**100**	83.3	0.83	75.0	83.3	66.7	0.61	70.8	75.0	66.7	0.64	76.7
**Random Tree**	83.3	**91.7**	75.0	0.83	70.8	66.7	75.0	0.71	70.8	66.7	75.0	0.71	75.0
**K star**	70.8	66.7	75.0	0.83	79.2	75.0	83.3	0.82	58.3	**100**	16.7	0.58	70.7
**LDA**	62.5	72.3	60.9	0.75	50.0	65.0	48.0	0.71	20.8	42.6	18.6	0.45	52.6

Acc: Accuracy, Sp: Specificity, Sn: Sensitivity, AUC: Area under ROC curve, Avg: Average score in % for each algorithms, *dnf:* Did not Finish”, * denotes Avg. from 3 significance levels. Measures >90% are marked in bold.

### Comparative analysis of worst time performance of classification algorithms over data sets

The amount of time taken by each algorithm to build the model and perform cross validation was measured. Table 9 shows the time in milliseconds for each algorithm at the lowest level of significance when the number of peptides nears 1000. Random Tree was the fastest, at ~1000 milliseconds (average) to complete the task, while MLP was the worst which did not finish due to high memory requirements. Random tree, Hyper Pipes, Naïve Bayes, VFI and KNN were the five fastest algorithms; each took less than ~4000 milliseconds to complete classification of >1,000 peptides. Logistic Regression and Attribute Selected Classifier, MLP were among the slowest algorithms taking more than 20 minutes to perform classification of >1,000 peptides. The absolute ranking for every algorithm was consistent per dataset; only three datasets have been considered to measure time performance.

**Table 9:** Worst case time performance (in ms) of classification algorithms

**Data set**	**Diabetes**	**Alzheimer’s**	**Antibodies**	**Avg. (in ms)**	**Rank**
**Random Tree**	**1809**	**491**	**1478**	**1260**	**1**
**KNN**	**3016**	**607**	**910**	**1511**	**2**
**Hyper Pipes**	**2486**	**602**	**2180**	**1756**	**3**
**Naïve Bayes**	**4780**	**1158**	**2480**	**2806**	**4**
**VFI**	**7440**	**1357**	**3000**	**3932**	**5**
**J48**	16581	**1385**	11731	**9899**	**6**
**K star**	25974	**2348**	6341	11555	**7**
**SVM**	10496	**2722**	29008	14076	**8**
**R. Forest**	50087	**8032**	21452	26524	**9**
**M5P**	50290	**8563**	23452	27435	**10**
**Bayes Net**	55672	**9031**	25000	29901	**11**
**K-means**	85955	12405	29658	42672	**12**
**SLR**	632840	48215	605365	428806	**13**
**LDA**	658668	869523	632983	720391	**14**
**Logistic R.**	1589092	1146783	1315256	1350377	**15**
**ASC**	5444533	2465021	4565896	4158483	**16**
**MLP**	*dnf*	*dnf*	*dnf*	*NA*	**17**

Table showing time performance in milliseconds over >1000 peptides for three datasets. Random Tree, KNN, Hyper Pipes and VFI were among the fastest. MLP were among the slowest with dnf: “Did not finish”. Time measurements less than 10 seconds are marked in bold.

### Comparative analysis of time performance of classification algorithms at different levels of significance over three data sets

For each level of significance, time was measured for each algorithm to build the model and for cross validation. At the highest level of significance (about 10 peptides), each algorithm were fast enough to complete the task in under 25 seconds. Execution times increased as the level of significance was lowered due to the higher number of features and increased difficulty in constructing the model. Table 10 shows classification algorithms time performance at various levels of significance.

**Table 10:** Time performance (in ms) of classification algorithms on datasets

	**Diabetes dataset**				**Alzheimer’s dataset**				**Antibodies dataset**			
**p value**<	**5x10^-13^**	**5x10^-10^**	**5x10^-7^**	**5x10^-4^**	**5x10^-5^**	**5x10^-4^**	**5x10^-3^**	**5x10^-2^**	**5x10^-8^**	**5x10^-7^**	**5x10^-6^**	**5x10^-5^**
**R. Tree**	**337**	**408**	**571**	**1809**	**184**	**200**	**218**	**491**	**250**	**265**	**608**	**1478**
**KNN**	**265**	**333**	**585**	**3016**	**130**	**156**	**239**	**607**	**187**	**234**	**414**	**910**
**Hyper Pipes**	**226**	**274**	**630**	**2486**	**119**	**259**	**423**	**602**	**281**	**312**	**736**	**2180**
**Naïve Bayes**	**250**	**456**	**1120**	**4780**	**182**	**340**	**500**	**1158**	**265**	**362**	**892**	**2480**
**VFI**	**299**	**561**	**1384**	**7440**	**187**	**337**	**623**	**1357**	**280**	**368**	**1379**	**3000**
**J48**	**415**	**833**	**3718**	16581	**166**	**256**	**712**	**1385**	**468**	**880**	**3011**	11731
**K star**	**468**	**1387**	**4150**	25974	**187**	**260**	**666**	**2349**	**299**	**562**	**2340**	**6341**
**SVM**	**3313**	**3635**	**5304**	10496	**1054**	**1108**	**1389**	**2722**	18297	18372	23712	29009
**R. Forest**	**5717**	11889	18254	50087	**952**	**1852**	**4843**	**8032**	**5004**	**6749**	13848	21452
**M5P**	**701**	**2583**	**7717**	50290	**290**	**524**	**2324**	**8563**	**2632**	**4711**	12033	23452
**Bayes Net**	**718**	**2087**	**5653**	55672	**334**	**662**	**4996**	**9031**	**733**	**1140**	**3394**	25000
**K means**	**2618**	**6651**	11876	85955	**593**	**1123**	**7212**	12405	**850**	**908**	**3442**	29658
**SLR**	11215	26380	79308	632840	**1330**	**3413**	22625	48215	17389	20649	89107	605365
**LDA**	**683**	**1044**	**7994**	658668	**402**	**699**	35568	869523	**1512**	**2018**	17373	632983
**Logistic R.**	**1204**	**2592**	24687	1589092	**629**	**1651**	48659	1146783	**1654**	**9379**	255103	1315256
**ASC**	**864**	**3504**	32836	5444533	**518**	**1859**	36849	2465021	**1217**	**1763**	25496	4565896
**MLP**	23759	314076	4572305	*dnf*	**2057**	30342	2789485	*dnf*	22916	156905	3277395	*dnf*

Table showing time performance in milliseconds on all level of significance for three datasets. MLP were among the slowest with dnf: “Did not finish”. Time measurements less than 10 seconds are marked in bold.

### Results summary

We have explored several disparate classifiers using a relatively new type of microarray data: immunosignaturing data. The tested algorithms come from a broad family of approaches to classify data. We chose algorithms from Bayesian, regression, trees, multivariate and meta analysis and we believe we have sampled sufficiently that the results are relevant. From Table 2 we found that Naïve Bayes had a higher average performance than all other algorithms tested. Naïve Bayes achieved > 90% average for 2 classes datasets where there is a clear distinction between two classes. For the multi-class the Antibodies dataset, where there is a clear difference between different types of antibodies, Naïve Bayes scored 88% average accuracy and was ranked third, close to the 93.3% accuracy of random forest. On the Asthma dataset, containing four classes, none of the algorithms were able to achieve more than 75% accuracy. This matches the biological interpretation very well. Naïve Bayes outperformed all algorithms for speed and accuracy, achieving 77.7% average score overall. Naïve Bayes was one of the top five fastest algorithms, ~500 times faster than the logistic regression. A summary of the all algorithms performance measures and time is given in below and described in Table 11. Distance metrics have been defined to access performance measures for all algorithms compared to the highest scoring algorithm on a given dataset.

I. Naïve Bayes: Naïve Bayes performed best overall with > 90% overall average score. It was always among the top 3 algorithms in all 7 comparisons. It ranked first 5 out 7 times when comparing all datasets. It was on an average just 0.3% behind the rank 1 algorithm in overall comparison. It is 2X slower than the fastest algorithm due to its mathematical properties. It would be feasible to perform large-scale classification studies using Naïve Bayes.

II. Multilayer Perceptron (MLP): It ranked second with overall score of 87.3% and was very close to SVM. The overall score is biased since MLP did not finish for level containing ~1000 peptides and hence scored was averaged from just the three levels. It was the slowest algorithm and infeasible to perform large-scale classification.

III. Support Vector Machines (SVM): Although it ranked third, it was not significantly different from the MLP in terms of performance measures. It was 700X faster than MLP and achieved >90% measured accuracy 3 times. Both MLP and SVM were <5% behind the rank 1 algorithm on average.

IV. VFI: VFI ranked fourth in overall performance measures and was the among top 5 fastest algorithms due to its voting method. Four times it obtained >90% average overall accuracy and ranked 2^nd^ twice.

V. Hyper Pipes: Hyper pipes ranked fifth overall in performance measures and was among the fastest of the tested algorithms, likely due to its inherently simplistic ranking method. It was <8% from first place 6 times.

VI. Random Forest: Random forest ranked sixth in overall performance measures and performed better on datasets having multiple classes (Antibodies and Asthma). It was 21 times slower than the fastest algorithm due to bootstrapping.

VII. Bayes net: Ranked in the middle for overall accuracy and time. It scored >90% overall measures twice. It was slower than the Naïve Bayes due to construction of networks in the form of an acyclic graph and it is relatively inefficient compared to Naïve Bayes due to the change in network topology during assessment of probability.

VIII. K means: K-means ranked eighth in overall performance measures and was 34X slower than the fastest algorithm in time performance due to the multiple iterations required to form clusters. It performed far better for 2 classes compared to multiple classes because guaranteed convergence, scalability and linear separation boundaries are more easily maintained.

IX. Logistic Regression: Logistic regression ranked ninth in overall accuracy. It was >90% three times. It was among the worst in time performance, being ~1000 times slower than the fastest algorithm as it needs to regress on high number of features. It is efficient for small numbers of features and sample sizes > 400.

X. Simple Logistic: It ranked tenth in overall performance measures and ranked first on the diabetes dataset. It ranked second in multiclass Asthma dataset. It was slow in time performance due to LogitBoost iterations.

XI. K nearest neighbors: It performed well on the 2 classes dataset but didn’t perform as well for multi class datasets. It was >90% performance for only rather difficult Diabetes dataset. This may be related to evenly defined but diffuse clusters related to the subtle differences between the Asthma patients.

XII. K star: It performed >90% for only the Diabetes dataset and was 9 times slower than the fastest algorithm. This algorithm may also be sensitive to the even and diffuse clusters described by this dataset.

XIII. M5P: It did not perform well on either time performance or accuracy. It never achieved >90% average score and was 22 times slower than the fastest algorithm due to formation of comprehensive linear model for every interior node of the unpruned tree.

XIV. J48: Top 5 fastest algorithm due to rapid construction of trees. It was >20% behind from the rank 1 algorithm on an average; its lower performance may possibly be due to formation of empty/insignificant branches which often leads to overtraining.

XV. Random Trees: It was the fastest algorithm since it builds trees of height log(k) where k is the number of attributes, however it achieves poor accuracy since it performs no pruning.

XVI. Attribute Selected Classifier (ASC): One of the slowest algorithms as it had to evaluate attributes prior to classification. It underperformed in performance measures due to the C4.5 classifier limitations that prevent overtraining.

XVII. Linear Discriminant Analysis (LDA): Its performance accuracy decreased as the number of features increased due to its inability to deal with highly variant data. It was slow (>500X slower than the fastest algorithm) since it tries to optimize class distinctions but the variance covariance matrix increases dramatically as the number of features increased.

**Table 11:** Summary of performance and time measures of classification algorithms

	**# Rank 1**	**# Rank 2**	**# >90%**	**Distance**	**Time**
**Naïve Bayes**	5	1	6	**−0.3**	**2X**
**MLP**	0	0	4	**−3.4**	7615X
**SVM**	0	1	3	**−3.6**	11X
**VFI**	0	2	4	−5.7	**3X**
**Hyper Pipes**	0	0	0	−7.9	**1X**
**R. Forest**	1	0	2	−8.8	21X
**Bayes Net**	0	1	2	−8.8	24X
**K-means**	0	0	1	−9.9	34X
**Logistic R.**	0	1	3	−11.8	1072X
**SLR**	1	1	2	−12.9	340X
**KNN**	0	0	1	−14.4	**1X**
**K star**	0	0	1	−16.5	9X
**M5P**	0	0	0	−17.0	22X
**J48**	0	0	0	−20.2	8X
**Random Tree**	0	0	0	−20.7	**1X**
**ASC**	0	0	0	−22.1	3300X
**LDA**	0	0	0	−24.0	572X

#Rank 1, Rank 2: No. of times algorithm ranked 1^st^ and 2^nd^ on 7 datasets, # > 90%: No. of times algorithm scored overall average score >90% on 7 datasets, Distance: magnitude an algorithm trails behind on average from the Rank 1 for the datasets (5% or less distance are marked in bold). Time: performance slower with respective to fastest algorithm. Time performances slower by 5 folds to fastest algorithm are marked in bold.

## Discussion

The comparisons provided in this article provide a glimpse into how existing classification algorithms handle data with intrinsically different properties than traditional microarray expression data. Immunosignaturing provides a means to quantify the dispersion of serum (or saliva) antibodies that result from disease or other immune challenge. Unlike most phage display or other panning experiments, fewer but longer random-sequence peptides are used. Rather than converging to relatively few sequences, the immunosignaturing microarray provides data on the binding affinity of all 10,000 peptides with high precision. Classifiers in the open-source program WEKA were used to determine whether any algorithm stood out as being particularly well suited for these data. The 17 classifiers, which were tested, are readily available and represent some of the most widely used classification methods in biology. However, they also represent classifiers that are diverse at the most fundamental levels. Tree methods, regression, and clustering are inherently different; the grouping methods are quite varied and top-down or bottom-up paradigms address data structures in substantially different ways. Given this, we present and interpret the results from our tests, which we believe will be applicable to any dataset with target-probe interactions similar to immunosignaturing microarrays.

From the comparisons above, Naïve Bayes was the superior analysis method in all aspects. Naïve Bayes assumes a feature independent model, which may account for its superior performance. It relies on the degree of correlation of the attributes in the dataset; for immunosignaturing, the number of attributes can be quite large. In gene expression data, where genes are connected by gene regulatory networks, there is a direct and significant correlation between hub genes and dependent genes. This relationship affects the performance of Naïve Bayes by limiting its efficiency through multiple containers of similarly - connected features [39-41]. In peptide-antibody arrays, where the signals that arise from the peptides are multiplexed signals of many antibodies attaching to many peptides, there is no direct correlation between peptides, but there is a general trend. Moreover, there is a competition of antibodies attaching to a single peptide, which makes it difficult for multiple mimotopes to show significant correlation with each other. Thus, the 10,000 random peptides have no direct relationships to each other each contributes partially to defining the disease state. This makes the immunosignaturing technology a better fit for the assumption of strong feature independence employed by the Naïve Bayes technique, and the fact that reproducible data can be had at intensity values down to 1 standard deviation above background enables enormous numbers of informative, precise, and independent features. Presence or absence of a few high- or low-binding peptides on the microarray will not impact the binding affinity for any other peptide, since the kinetics ensures that the antibody pool is not limiting. This is important when building microarrays with >300,000 features per physical assay, as in our newest microarray. More than 90% of the peptides on either microarray demonstrate normal distribution for binding signals. This is important since feature selection methods used in this analysis (*t*-test and one way ANOVA) and the Naïve Bayes classifier all assume normal distribution of features.

The Naïve Bayes approach requires relatively little training data, which makes it a very good fit for the biomarker field. The sample sizes usually range from N = 20-100 for the training set. Naïve Bayes has other advantages as well: it can train well on a small but high feature data set and still yield good prediction accuracy on a large test set. Any microarray with more than a few thousand probes succumbs to the issue of dimensionality. Since Naïve Bayes independently estimates each distribution instead of calculating a covariance or correlation matrix, it escapes relatively unharmed from problems of dimensionality.

The data used here for evaluating the algorithms were generated using an array with 10,000 different features, almost all of which contribute information. We have arrays with >300,000 peptides per assay (current microarrays are available from http://www.peptidearraycore.com) which should provide for less sharing between peptide and antibody, effectively spreading out antibodies over the peptides with more specificity. This presumably will allow resolving antibody populations with finer detail. This expansion may require a classification method that is robust to noise, irrelevant attributes and redundancy. Naïve Bayes has an outstanding edge in this regard as it is robust to noisy data since such data points are averaged out when estimating conditional probabilities. It can also handle missing values by ignoring them during model building and classification. It is highly robust to irrelevant and redundant attributes because if Y_i_ is irrelevant then P (Class|Y_i_) becomes uniformly distributed. This is due to that fact that the class conditional probability for Xi has no significant impact on the overall computation of posterior probability. Naïve Bayes will arrive at a correct classification as long as the correct classes are even slightly more predictable than the alternative. Here, class probabilities need not be estimated very well, which corresponds to the practical reality of immunosignaturing: signals are multiplexed due to competition, affinity, and other technological limitation of spotting, background and other biochemical effects that exist between antibody and mimotope.

### Time efficiency

As the immunosignaturing technology is increasingly used for large-scale experiments, it will result in an explosion of data. We need an algorithm that is accurate and can process enormous amounts of data with low memory overhead and fast enough for model building and evaluation. One aims for next-generation immunosignaturing microarrays is to monitor the health status of a large population on an on-going basis. The number of selected attributes will no longer be limited in such a scenario. For risk evaluation, complex patterns must be normalized against themselves at regular intervals. This time analysis would require a conditional probabilistic argument along with the capacity of accurately predicting the risk with low computational cost. The slope of Naïve Bayes on time performance scale is extremely small, allowing it to process a large number of attributes.

## Conclusion

Immunosignaturing is a novel approach which aims to detect complex patterns of antibodies produced in acute or chronic disease. This complex pattern is obtained using random peptide microarrays where 10,000 random peptides are exposed to antibodies in sera/plasma/saliva. Antibody binding to the peptides is not one-to-one but a more complicated and multiplexed process. The quantity and appearance of this data appears numerically, distributionally, and statistically the same as gene expression microarray data, but is fundamentally quite different. The relationships between attributes and functionality of those attributes are not the same. Hence, traditional classification algorithms used in gene expression data might be suboptimal for analyzing immunosignaturing results. We investigated 17 different kinds of classification algorithm spanning Bayesian, regression, tree based approaches and meta-analysis and compared their leave-one-out cross-validated accuracy values using various numbers of features. We found that the Naïve Bayes classification algorithm outperforms the majority of the classification algorithms in classification accuracy and in time performance, which is not the case for expression microarrays [42]. We also discussed its assumptions, simplicity, and fitness for immunosignaturing data. More than most, these data provide access to the information found in antibodies. Deconvoluting this information was a barrier to using antibodies as biomarkers. Pairing immunosignaturing with Naïve Bayes classification may open up the immune system to a more systematic analysis of disease.

## Competing interests

US Patent Compound Arrays for Sample profiling: 61218890, US Patent ‘Naïve Bayes Classification for Immunosignaturing M12-104L, SAJ is cofounder of HealthTell Diagnostics which owns the patent to immunosignaturing.

## Authors’ contributions

MK completed the analysis of all data, and the original manuscript draft. PS completed all revisions and consulted on analysis. SAJ/PS co-invented immunosignaturing, SAJ funded the project. All authors read and approved the revised manuscript.

## Ethics statement

Consent was obtained for every sample in this manuscript and was approved by ASU IRB according to protocol number 0912004625 entitled "Profiling Human Sera for Unique Antibody Signatures". Humans were consented by the retrieving institution and a materials transfer agreement was signed between the Biodesign Institute and the collaborating institute. The collaborating institutes' protocols were current and each human subject signed an approved consent form and released their sera.
